# Horizon scanning implanted biosensors in personalising breast cancer management: First pilot study of breast cancer patients views

**DOI:** 10.1002/hsr2.30

**Published:** 2018-03-15

**Authors:** Theresa Ikegwuonu, Gill Haddow, Joyce Tait, Alan F. Murray, Ian H. Kunkler

**Affiliations:** ^1^ Innogen Institute The University of Edinburgh Edinburgh Scotland; ^2^ Science, Technology and Innovation Studies The University of Edinburgh Edinburgh Scotland; ^3^ Institute for Bioengineering, School of Engineering University of Edinburgh Edinburgh Scotland; ^4^ Edinburgh Cancer Research Centre, Institute of Genetic and Molecular Medicine Western General Hospital Edinburgh Scotland

**Keywords:** cancer, oncology, adjuvant radiotherapy, breast cancer, biosensor, implantable technology, patient acceptability

## Abstract

**Aims:**

This study aimed to explore breast cancer patients' understanding and acceptability of implanted biosensors (BS) within the primary tumour to personalise adjuvant radiotherapy, and to determine optimal design and number of BS, and evaluate potential clinical benefits as well as concerns about tolerance, toxicity, dwell time, and confidentiality of data.

**Patients and methods:**

A total of 32 patients treated by surgery (29 breast conserving, 3 mastectomy), postoperative radiotherapy and systemic therapy for early breast cancer, were recruited from a posttreatment radiotherapy clinic at a cancer centre. Patients participated in semistructured interviews. Interview transcripts were analysed using qualitative methods.

**Results:**

Participants were aged 39 to 87 years, with a median age of 62 years. Most (N = 23[72%]) were unfamiliar with biosensors. The majority (N = 29[90.6%]) were supportive of the technology's potential use in future breast cancer treatment and were willing to accept biosensors (N = 28[88%]) if they were endorsed by their breast cancer consultant. Only 3 patients expressed concerns, predominantly about uncertainties on their role in the diagnostic and treatment pathway. Patients were flexible about the size and shape of BS, but had a preference for small size (N = 28 [87.5%]). Most (N = 22[69%]) would accept implantation of more than 5 BS and were flexible (N = 22[69%]) about indefinite dwell time. Patients had a strong preference for wireless powering of the BS (N = 28[87.5%]). Few had concerns about loss of confidentiality of data collected. All patients considered biosensors to be potentially of important clinical benefit.

**Conclusions:**

While knowledge of biosensors was limited, patients were generally supportive of biosensors implanted within the primary tumour to collect data that might personalise and improve breast cancer radiotherapy in future.

## 1 INTRODUCTION

Radiotherapy uses ionising radiation, normally delivered by treatment machines (linear accelerators, LINACs), to kill or control cancer cells by damaging their DNA. If radiotherapy is given to the organ containing cancer after surgery, to kill any residual cancer cells, it is called “adjuvant.” Radiation beams can be shaped to treat the organ (for example, the breast) once the cancer has been removed by surgery while minimising dosage to other organs such as the heart. Prior to a course of radiotherapy, a dose “map” is created to show the distribution of the X‐ray dose within the breast. Typically for breast cancer, after surgery, small daily doses of radiation based on this dose “map” are delivered to the patient lying on the treatment couch by an LINAC over 3 to 4 weeks.^1^ However, each dose is not adjusted to the changing biology of the cancer during treatment. There is now increased scientific interest in adapting radiotherapy to the biology of the individual patient and his/her tumour to improve clinical outcomes.

Adjuvant radiotherapy remains, along with surgery and systemic therapy, a cornerstone of the treatment of early breast cancer. Adjuvant radiotherapy roughly halves the risk of first recurrence after breast conserving surgery^2^ and improves survival rates both after breast conserving surgery^2^ and mastectomy.^3^ Selection for adjuvant radiotherapy after mastectomy is based on clinico‐pathological factors (eg, tumour size, and spread to local lymph nodes). However, there are no reliable measures (biomarkers) of a tumour's likely response to radiotherapy to predict which patients with cancer in general, or breast cancer in particular, are likely to respond to radiotherapy.^4^ There is increasing interest in treating breast tumours before surgery, where the intact tumour can be used to monitor response. This approach is well established for hormonal and chemotherapy to shrink breast cancers before surgery.^5^, ^6^ It is, however, possible in the future, that radiotherapy might be combined with anticancer drug treatment in this preoperative setting. A small study has shown that preoperative radiotherapy in combination with chemotherapy does not compromise subsequent breast conserving surgery.^7^

Biosensors are analytic devices that measure biological characteristics of tissue and body fluids.^8^ These may be physical parameters such as O_2_ concentration and local pH, or more specific biomarkers such as Caspases.^9^ The leading example of the use of biosensors in health care is to monitor diabetes. This point of care testing has enabled patients to self‐test and manage their own diabetes with substantial reduction in global health care costs.^10^

**Figure 1:**
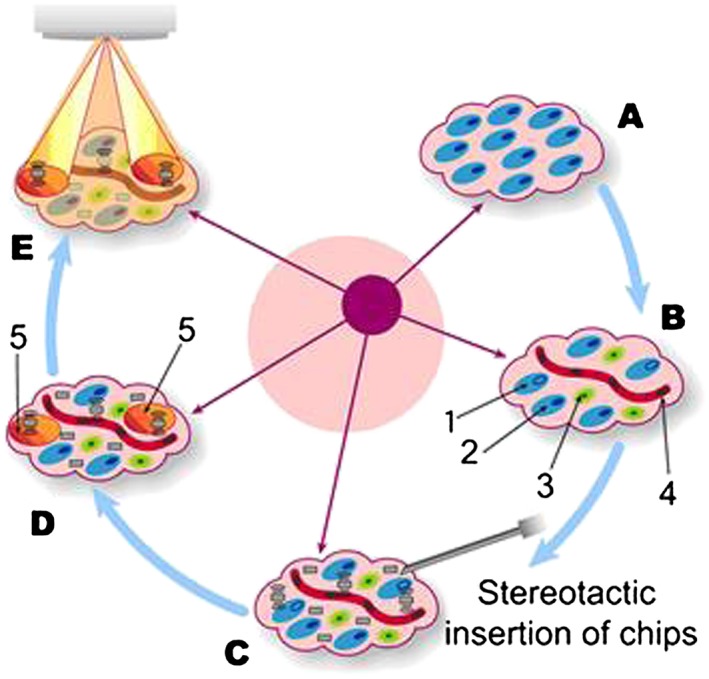
Biosensors in radiation treatment of cancer. A, Group of cancer cells. B, Radio‐resistant hypoxic (1) and radiosensitive (2) cells with immune (3) and vascular (4) cells in the tumour microenvironment. C, Stereotactic insertion of biosensors into the microenvironment. D, Signal output (5) from biosensors in hypoxic radio‐resistant zones. E, Differential deposition of radiation treatment to hypoxic radio‐resistant zones

The tumour microenvironment is the cellular setting in which the cancer exists. It includes blood vessels, immune cells, and inflammatory cells (see Figure 1). The development of biosensors for real‐time monitoring of the tumour microenvironment offers the promise of clinical dividends, by connecting an individual tumour's biology to personalised cancer treatment.^11^ In addition, chemical biosensors can be implanted into experimental tumours to detect levels of specific biomarkers.^12^ Peptide‐based sensors, using a previously related trypsin sensor as a model, have been reported.^13^These may provide a platform for the measurement of specific markers of interest such as the caspase family of enzymes for cell death. Miniaturised versions of the ISFET^14^ (ion‐sensitive field effect transistor) and Clark Electrode sensors^15^ provide the basis for measuring tumour hypoxia (low levels of oxygenation) continuously.

Tumour hypoxia is an important factor in both resistance to radiation and chemotherapy.^16^, ^17^ Radiotherapy requires oxygen to fix radiation damage. It is estimated that about 40% of all breast tumours and 50% of advanced breast cancers contain hypoxic regions.^18^ In cancer tumours, demand for oxygen often exceeds supply. The abnormal and chaotic vasculature of solid tumours can result in temporal and spatial changes in hypoxia.^19^, ^20^ However, there is currently no clinically applicable method of measuring these spatial and temporal changes in hypoxia in real time, to use this information to the dose distribution “map” to selectively increase the dose to hypoxic areas during a course of radiotherapy.

Developing real‐time biosensors of breast cancer biology to individualise radiotherapy has been identified as a research priority.^21^ A cross‐disciplinary project funded by EPSRC, IMPACT (Implantable Microsystems for Personalised Cancer Therapy) is currently developing a wireless silicon platform^22^ to sense real‐time changes in tumour biology through measurement of pH, oxygenation, and cell death. It is envisaged that in the future, subject to ethical and other necessary approvals and validation in preclinical and clinical studies, a number of biosensors will be inserted into regions of tumour hypoxia. Sensing hypoxia in real time may allow extra radiation dose to be delivered differentially to hypoxia areas (see Figure 1) during a course of curative radiotherapy, to improve tumour response and cure rates.^23^

There is a dearth of published literature on the views of cancer patients on the risks and benefits of tumour biosensors to personalise radiotherapy treatment.^24^ Little is known about patient willingness to accept, albeit hypothetically, implanted devices to improve the outcome of treatment. It is important to assess acceptability of novel health care devices among patients at a very early stage to guide future development, clinical research, and subsequent implementation into routine practice.

We report, to our knowledge for the first time, the views of breast cancer patients about the development of real‐time biosensors for individualised radiotherapy for early breast cancer.

## 2 METHODS

### 2.1 Recruitment

The study adopted a pragmatic approach to obtain a sample of patients with early breast cancer treated by adjuvant radiotherapy after surgery. Patients were recruited based on cancer type and cancer treatment. Potential participants were initially identified and screened against the eligibility criteria by 4 consultant clinical oncologists specialising in breast cancer at the postradiotherapy review clinic at the Edinburgh Cancer Centre.

Inclusion criteria were adults with a diagnosis of operable early breast cancer treated by breast conserving surgery or mastectomy, who had completed their anticancer treatment. Patients who had locally advanced or metastatic disease or were unable to consent or take part in an interview, or those vulnerable/unsuitable as screened by the consultants, were excluded from the study. Women were asked during their consultation at their postoperative radiotherapy review whether it was acceptable for the researcher to approach them in the waiting room. If the response was positive, the researcher offered a Participant Information Leaflet (PIL; see Appendix S2) and consent form as they were leaving. If the consent form was not returned, no follow‐up could be made. Over a 12‐week period in 2014, 32 women were recruited to the study, representing 73% of patients who were approached to participate. Patients who declined to participate were not required to give any reason.

This research was granted ethical approval from National Health Service Ethics Committee (REC reference number 10/S1103/41). Participants provided written informed consent prior to being interviewed. Participation in the study was on a voluntary basis, and no incentives were offered.

Permission to record the interview was gained to have an accurate record of the discussion. Most interviews were performed in patients' homes (N = 28) and a few at the cancer centre (N = 4), based on patients' preferences. Interviews were recorded by the researchers [TI/GH] with a digital voice recorder and uploaded to a secure folder on a University of Edinburgh hard drive. They were transcribed and anonymised by the researcher [TI]. Transcripts were analysed using qualitative data analysis software package QSR Nvivo 10 following a standard grounded theory approach, using a broadly inductive approach, ie, conclusions were directly driven by the data. Initially, emerging patterns and themes were captured with an open‐coding method. Following this, they were grouped into broader coding categories and then further refined into more abstract themes. Researchers' backgrounds were in the social, medical, and engineering sciences, covering a range of expertise in research methods and approaches. Differing viewpoints were discussed, and reliability of coding was achieved through consensus agreement of the researchers. An iterative pattern of data collection, reflection/analysis, further data collection, further reflection and analysis, and finally synthesis was employed.^25^, ^26^

The interview focussed on patients' views regarding the use of implanted biosensors and included possible technological issues related to the development of biosensors (eg, different shapes, sizes and insertion techniques, data transfer, and data security); see Appendix 1. The interviews also explored risks (eg, infection), issues around insertion, and the dwell time for which it was acceptable to retain the sensor. Patients were presented with standardised written information about implanted biosensors within the primary tumour and their potential clinical value (eg, monitoring the cancer's biology and taking real‐time measurements to individualise future radiotherapy treatment).

**Figure 2:**
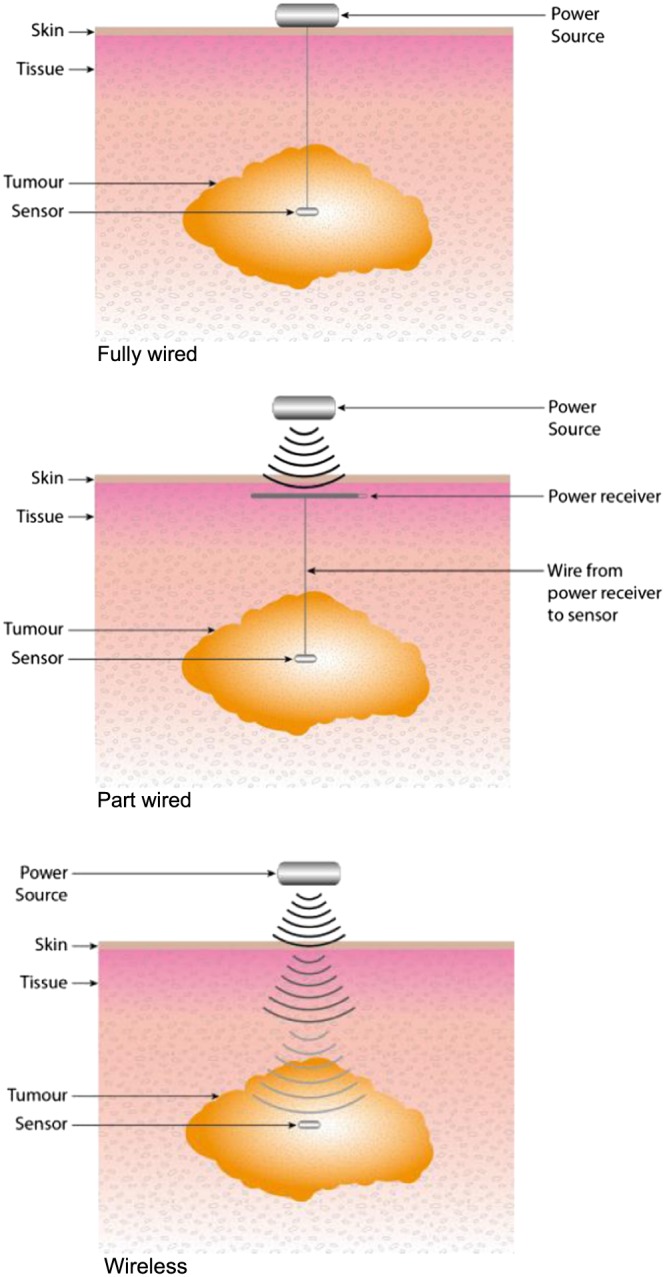
Example of stimulus material shown to participants (options to power the biosensors)

During the interviews, all patients were shown medical illustrations (see Figure 2 as an example), illustrating actual models of biosensors, showing their sizes, shapes, and powering options. The medical illustrations explained how the biosensor/s would be inserted and showed 3 options for power and data transmission (wired, part‐wired, and wireless). Patients were then asked about their preferences in relation to size and shape of the biosensors, as well as power and data transmission options. Following good practice in qualitative data collection methods, a process of review and analysis was applied, and a pilot interview was performed to test the interview schedule in terms of patients' understanding and order of questions.^27^

## 3 RESULTS

Overall, 32 patients took part in a semistructured interview, which lasted approximately 1 hour. All participants were adults, female, English‐speaking patients who had completed their anticancer treatment.

### 3.1 Patient demographics and breast cancer experience

Participants were aged between 39 and 87 years; mean age was 62 years. Two thirds of participants were married or had a partner, and one third, either widowed or separated. The majority (N = 24, 75%) had children (and grandchildren). In relation to how the breast cancer was detected, there was an almost equal split between self‐referral/finding a lump and screening mammography (Table 1). The majority (N = 25, 78.1%) had been treated by surgery adjuvant radiotherapy and hormonal therapy after breast conserving surgery. Only 3 (9.4%) had undergone a mastectomy, while 21.9% of patients (N = 7) had received chemotherapy (see Table 1).

**Table 1:** Demographic, clinical presentation, and treatment features of the study population

Participants in Sample (n = 32)	Frequency	Percentage
Age		Range
Mean	62 y	39‐87 y (SD = 11.6)
<50 y	3	9.4%
51‐60 y	10	31.2%
61‐70 y	10	31.2%
>60 y	9	28.2%
Gender		
Female	32	100%
Marital status		
Married/partner	22	68.8%
Widowed/ separated/single	10	31.2%
Family status		
Children/grandchildren	24	75%
No children/grandchildren	8	25%
How was breast cancer found		
Screening mammography	17	53.1%
Found lump/ self‐referral	15	46.9%
Breast cancer treatment received		
Surgery (breast conserving) and radiotherapy	25	78.1%
Chemotherapy, surgery, and radiotherapy	6	18.7%
(breast conserving N = 4, mastectomy N = 2)		
Chemotherapy and surgery (mastectomy)	1	3.2%
Total	32	100%

### 3.2 Familiarity with the concept of biosensors

Most patients (N = 23, 72%) said they had not heard of the term “biosensor” prior to taking part in this project. Almost one third (N = 9, 28%) thought they had heard the term or had an idea what it was.

### 3.3 Patients' general views about biosensors

Of the 32 patients interviewed, almost all (n = 29) were in favour of allowing a biosensor to be used during treatment.

“I suppose, we're talking about potentially saving your life. I don't see why anybody would not want to have whatever you need to have in order to get a result.” (Participant 9)

Most patients (N = 29, 90.6%) suggested that they would be supportive of the biosensor technology. Patients tended to be supportive of the biosensor technology if endorsed and recommended by their breast cancer consultant:

“Yes, I would use it if my doctor suggested it.” (Participant 23)

Perceived benefits went beyond the individual patient and often included family members or the wider group of breast cancer patients who may benefit from the biosensor technology in future:

“I think if something's going to help and it's going to help others in the future, that's the main thing.” (Participant 1)

### 3.4 Patients' concerns about biosensors

Only a small number (N = 3, 9.4%) reported uncertainty about whether they would accept a biosensor in future. Mainly, they were unconvinced with how it would fit within the framework of their treatment pathway:

“See that's quite a hard one to answer in my particular case, because from diagnosis to surgery was only two and a half weeks, so, you know, would there even have been time to do anything in my case, I don't know?” (Participant 11)

Patients acknowledged the discomfort associated with inserting the biosensors and the potential for complications, such as an infection, allergic reaction or an embolism. Approximately, half of patients (N = 18, 57%) stated that they would want to know more about the likelihood of a complication or risk occurring.

“I would want to know what the percentages were, so that [...] you can make an informed decision.” (Participant 21)

### 3.5 Size and shape

When discussing the potential size of biosensors, patients tended to accept any size. Their focus was on “whatever helps to get better,” reiterating the theme of recovery from cancer. However, many (N = 28, 88%) did express a preference for a smaller size, perceived as less intrusive and less painful, especially during insertion.

However, there was a “once it is in, what difference does the shape make?” philosophy expressed; 54% (N = 17) either did not mind or were unsure about their preference in relation to shape.

When preferences were expressed, 34% of patients placed emphasis on a smooth surface with a long shape and rounded edges:

“Instinctively when it's got a curved end you think it'll be less sore going in.” (Participant 7)

Although pain on insertion seemed to affect views, again, emphasis was placed on the benefits of using biosensors and recovery:

“I suppose when you actually think about the process you've already gone through, and you had the needles with the biopsies and everything else, if it's going to cure me or help me get rid of cancer. […] I would say yes, go for it.” (Participant 5)

### 3.6 Number of biosensors

There was a range of views expressed in relation to the number of biosensors that may be inserted. However, the majority (N = 22, 69%) expressed a willingness to accept more than 5 biosensors, if this was required for their treatment.

A small number of patients expressed concerns about having a higher number of biosensors inserted:

“I think I'd draw the line at probably three, I would think. But again, it would depend on the tumour.” (Participant 27)

Yet most patients would agree to have more than 1 biosensor inserted if this was necessary for their treatment. This view was based on trust in their health care professional and a focus on recovery.

### 3.7 Duration of biosensors in the body

Patients tended to be pragmatic about the dwell time the biosensor/s were left in the body. If it was small enough, and “it was doing its job” (Participant 7) and the biosensor could not be felt, then most (N = 22, 69%) were unconcerned about leaving the biosensor in:

“As long as they're not doing any more damage, […] you wouldn't feel them or anything so, yeah, I'd be happy for them to be there.” (Participant 11)

Trust in health care professionals could sometimes override patients' individual opinion and preference:

“I would just go on whatever the doctors recommended. […] if they said you need to keep it in for 3 months or six months or six years or like with the Tamoxifen, for example, I'm not happy to take that for ten years, but that's what I'm told to do, so that's what I'm going to do.” (Participant 9)

A minority were wary with having something “foreign” inside them, and 6 patients (18.5%) expressed a preference to have the biosensor removed once treatment had been completed:

“It's a foreign body, and it's not really meant to be there. And if it's there, it's there for a reason, and if that reason is now redundant then there would be no need for them. […] if it was the option to remove them, then I would probably want it removed.” (Participant 31)

### 3.8 Power and data transmission options

If a preference was expressed on power and data transmission options, then most patients (N = 28, 87.5% and N = 29, 90.6%, respectively, Table 2) favoured a wireless option. Wireless was perceived to be more convenient, advanced, and less visible on the surface of the skin. Patients also considered the practical implications of having visible wires on the surface of the skin. The wired option was perceived as more obtrusive, especially when getting dressed. It seemed important not to have anything visible on the surface of the skin. This was seen as a way of coping with cancer treatment.

“Option three [wireless] certainly seems to be the best one, because then you can always forget it's there, you see. And for me personally, for survival, I think it's good […] that you do not dwell on it too much when you have cancer.” (Participant 27)

**Table 2:** Power and data transmission choices

Option	Power (N = Number of Patients)	Data Transmission (N = Number of Patients)
Unsure	1	1
Fully wired	1	0
Part wired	1	3
Wireless	29	28

### 3.9 Patients' concerns about data security

In general, patients did not express concern around data security. This was either because patients assumed that data security was taken care of by the National Health Service and the medical professionals, or they did not believe that the data could be of interest and use to anyone else. Some compared the data with their personal financial information, concluding that readings from the biosensors would be a lot less valuable and interesting to other people:

“I use a bit of data security when I'm talking about my bank, but when I'm talking about the status of my tumour, I don't care who knows.” (Participant 12)

In most cases, patients were looking at the “bigger picture” and their main concern was on getting the best treatment available and being able to recover:

“I wouldn't worry about it [data security]. When you're going through cancer itself, you wouldn't be thinking about that; you'd just be thinking of how good this sensor and technology is that it can actually give a reading; and it's for your benefit.” (Participant 25)

### 3.10 Perceived importance of biosensor technology

All patients felt that the biosensor technology was important for the potential benefit of future cancer patients.

## 4 DISCUSSION

We present, to our knowledge, the first evidence of support for the potential future use of implanted biosensors from patients recently treated for early breast cancer. Implanted biosensors to monitor the tumour microenvironment may have wider application to other hypoxic tumours (brain, head and neck, lung, oesophageal, and prostate cancer).^28^ Previous research relating to implantable cardiac devices has found that in general, patients accepted their devices. However, they emphasised the importance of assessing patient acceptance to improve clinical outcome.^29^ Haddow and colleagues^30^ explored patients' reactions to implantable smart technologies, and highlighted that consideration needs to be given to how these technologies affect patients. Thus, it is vital to consider patient acceptability of novel implantable technologies to improve future cancer treatment outcomes.

It is recognised that the public perception and understanding of science are important to adoption of technological advances. In addition, both scientists and governments may misunderstand public concern about science and technology.^31^, ^32^ If public concerns are misinterpreted, this may result in public dissatisfaction.^33^ These arguments equally apply to advances in health technologies in general, and cancer care in particular.

New medical technologies in oncology (as well as other diseases) require endorsement from patients, as well as cancer professionals and regulatory bodies assessing safety and clinical efficacy. For example, concerns about safety have adversely affected public attitudes to the use of nanotechnology.^34^, ^35^

We believe our sample of breast cancer patients, albeit modest as a pilot study, is reasonably representative of patients treated by surgery and postoperative radiotherapy for early breast cancer. Over 75% of patients had been treated by breast conserving surgery and postoperative radiotherapy, the most common treatment policy for early breast cancer. We, therefore, think the sample was representative of standard care. Most patients had received some form of adjuvant systemic therapy. It has been our local policy in patients requiring adjuvant chemotherapy for this to be performed after surgery but before radiotherapy. There is normally 1 postradiotherapy visit (6‐8 wk after the completion of radiotherapy) before patients are referred back to surgical unit for annual follow‐up. Hence, review in the postradiotherapy clinic, the setting for this study, involved patients who had relatively recently completed and recovered from their postoperative radiotherapy.

Qualitative research is valuable as it increases understandings around why individuals would be willing to accept a technology. As this study also reiterates findings from earlier work with men recovering from prostate cancer,^24^ a degree of confidence regarding the validity, transferability and reliability of the present data, can be assumed.^36^

It is encouraging that most patients (29/32) [90.6%] were in favour of the idea of biosensors being implanted to improve breast cancer treatment. The endorsement of their cancer specialist was a strong factor influencing this view. This is consistent with the trust that breast cancer patients in general invest in the views of their specialist clinicians over the best treatment for them.^37^ Of note, patients considered the benefits not just to themselves but to the wider population of breast cancer patients. This is consistent with altruism being an important factor in participation in cancer clinical trials.^38^, ^39^ We need to be cautious in interpreting support among patients for biosensors for a number of reasons. First, the technology is at a very early stage of development and in advance of any prototype, so the medical illustrations of the size and shape of the biosensors and how many biosensors might be inserted are putative. Secondly, as with any health care technology, there is a trade‐off between clinical benefits and risks. We were not able to present any specific information to patients about the type and frequency of side effects of implanting the devices (pain, infection, biofouling, malfunction) and benefits (reduced risk of recurrence, improved survival) for monitoring the biology of their breast cancer for treatment purposes. Changes in the risk/benefit ratio, once this information is available in the future from early phase clinical trials, are likely to be more informative to patients. Other factors such as the requirement for a separate general anaesthetic, with associated, albeit small risks, might also reduce patient support. However, the main purpose of the study was to try and explore at an early stage in the development whether patients identified major concerns that have to be addressed before early phase trials and clinical implementation could be considered. We can envisage that in the future the use of biosensors of hypoxia may add valuable information to the radiotherapy planning “map.” This would allow the distribution of radiation energy to be adapted to the changing pattern of hypoxia during a course of radiotherapy treatment of breast cancer and other hypoxic tumours.

It is useful to know that patients expressed a strong preference for biosensors that worked wirelessly, with no protruding wires from the device to the surface of the skin. This is in line with other devices such as cardiac pacemakers that are placed subcutaneously to detect abnormal cardiac rhythms to maintain cardiac function. These are widely accepted by patients.^29^ Some patients in our study may have been aware of the availability of a variety of wearable devices to measure, for example, heart rate. These devices collect physiological data in real time so patients tended to be familiar with wearable devices. The step to measure the biological activity of their cancer might not be too great a conceptual leap. However, we did not question patients about their knowledge of wearable devices.

There was a limited number of patients who felt uncertain whether they would accept a biosensor as part of their treatment. However, their concerns were not related to risk about the device but to uncertainty about how the biosensor might fit into the patient treatment pathway. This is not surprising because it is too far from clinical implementation to predict how biosensors will fit into the treatment framework.

It is perhaps surprising that patients did not express more concern about confidentiality. This might be because they assumed that confidentiality would be maintained if the device was approved by NHS regulatory bodies. Cybersecurity is a legitimate concern with the risk of ill‐intentioned individuals or organisations hacking into IT used by the device, stealing confidential information or causing the device to malfunction. An increase in NHS cybersecurity breaches could adversely affect support from both patients and cancer professionals for biosensors in the management of breast cancer and other hypoxic cancers.

Willingness to accept implanted biosensors is not gender specific, as previous research with male participants,^24^ and this study with female participants have shown that both men and women recovering from cancer express some degree of acceptance. However, the impact of having recovered from cancer on this willingness to accept is not clear. Further research with other subgroups of the population may be required.

### 4.1 Study limitations

We recognise there are some limitations of this pilot study. The information presented to the patients on the size and shape of the biosensors is conjectural, since the biosensors are still in the design phase prior to testing in murine and veterinary tumour models to show “proof of principle.”^22^ It is likely that their eventual size, shape, and optimal number will be determined by further preclinical and clinical research. We recognise that preoperative radiotherapy in breast cancer remains investigational and may or may not become part of standard care in the future. It is possible, therefore, that the use of biosensors to individualise radiotherapy planning based on real‐time spatial and temporal measurements of hypoxia may find its initial applications in other solid hypoxic tumours (eg, brain, head and neck, lung, oesophagus, prostate, and cervix), in which radical radiotherapy with or without systemic therapy is the primary treatment where the tumour has not been removed surgically.

To explain the insertion of biosensors, we did use the analogy of patients having titanium clips inserted at the time of diagnostic biopsy or after breast conserving surgery to mark the tumour bed. However, as yet, the exact method of insertion is still at a research and development level preclinically.

This study only included patients who had completed their anticancer treatment, which means that the views of patients still undergoing treatment were not captured. Moreover, this study was performed in a single location. These limitations may have implications for the generalisability of the sample.

This study was not designed for and it was not a questionnaire of a representative sample to infer associations between demographics, and considering the sample size and single location of the study, inferential statistics were not performed. It might be worth doing a study designed specifically for that in the future.

### 4.2 Clinical implications

The development of electrochemical biosensors for real‐time monitoring of tumour microenvironment has potentially promising clinical dividends by connecting individual patient biology to individualised cancer treatment.

## 5 CONCLUSION

We can conclude from this study of patients' views of biosensors at a very early stage of development that none of the technology's potential downsides would preclude their further development for radiotherapy treatment of breast cancer. This helps justify the costs of continuing to invest in further research and development towards early clinical testing of biosensors for breast cancer and other solid tumours. If preclinical and clinical “proof of principle” is demonstrated in the IMPACT study,^22^ larger scale studies of patients' views at a more advanced stage of this novel technology will be needed to confirm our findings.

## CONFLICTS OF INTEREST

We have no conflicts of interest to disclose.

## FUNDING

This study was funded by the Engineering and Physical Sciences Research Council project number EP/K034510/1. During writing up, Theresa Ikegwuonu was supported by the UK Medical Research Council as part of the Informing Healthy Public Policy programme [MC_UU_12017/15] at the MRC/CSO Social and Public Health Sciences Unit, University of Glasgow. The UK MRC had no role in the design, collection, analysis, or interpretation of this study.

## AUTHOR CONTRIBUTIONS

Conceptualization: Theresa Ikegwuonu, Gill Haddow, Joyce Tait, Alan Murray, Ian Kunkler

Investigation: Theresa Ikegwuonu, Gill Haddow, Ian Kunkler

Methodology: Theresa Ikegwuonu, Gill Haddow, Joyce Tait, Alan Murray, Ian Kunkler

Formal analysis: Theresa Ikegwuonu, Gill Haddow

Funding acquisition: Alan Murray, Gill Haddow, Joyce Tait, Ian Kunkler

Writing – original draft: Theresa Ikegwuonu

Writing – review and editing: Gill Haddow, Joyce Tait, Alan Murray, Ian Kunkler, Theresa Ikegwuonu

## Supporting information

Appendix S1. Supporting information item

Appendix S2. Supporting information item
