# Supplementary material for: Horizon scanning implanted biosensors in personalising breast cancer management: First pilot study of breast cancer patients views
Source: Health Sci Rep. 2018 Mar 15;1(4):e30. doi: 10.1002/hsr2.30 (PMC6266376; doi:10.1002/hsr2.30)
Supplement: Supplementary file 1 — Appendix S1. Supporting information item [file HSR2-1-e30-s001.doc]

**Interview Schedule, V.5**

Interview No: Interview ID: BIOS_BC_

Date of Interview: Interviewer:

Pseudonym used:

**Pre- interview/ Introduction:**

My name is (Haddow/Ikegwuonu) and along with my colleague (Haddow/Ikegwuonu), we are social science researchers working at the University of Edinburgh. We are working with cancer specialists at the Western General Hospital, Edinburgh who are especially interested in what you think about ‘implanted biosensors’. We know that you will not have thought about this before or maybe not even have heard the word ‘biosensor’ before, but we will be able to talk you through what they are and show you what they may look like. We will be talking with thirty other individuals like your self who may have undergone treatment for cancer in the past about their views on this future technology.

So I am hoping we can chat for about 45 minutes to 1 hour (check if ok timewise). I do need to record our conversation but this is purely for accuracy of recall. I will download the audio recording which will be anonymised to protect your identity, before sending it to a trusted Innogen administrator who will transcribe it. The recording will be stored and treated confidentially in accordance with the Data Protection Act.

We really need to get your views on the development of this technology – both positive and negative (there are no right or wrong answers). What we need to know is what you think; your honest answers. We also appreciate that you may find it difficult to think about a device whose shape and size has not been finalised.

Would you like us to send you a short report about what we find? Is there anything you want to ask before we begin?

What made you want to take part in this research project?

**SECTION A: Information about Interviewee:**

Can I ask WHEN you were born?

Can I ask you where you were born?:

Are you married?:

Do you have any children?:

Do you or have you worked?

Have you any formal qualifications (PROBE: biology, science, etc)

**SECTION B: ICE-BREAKERS:**

Can we talk a little bit about your cancer treatment? Could you tell me about what treatment you received?

(If radiotherapy treatment received) Tell me about your radiotherapy treatment?

- More specific questions about radiotherapy sessions (when started; how long; etc.)

Descriptions of radiotherapy treatment?

General views on (radiotherapy) treatment (time, comfort, side effects/ impact on everyday life)

If you had breast conserving surgery, did you have any metal markers inserted at the time of surgery?

Did you have (or are you going to have) any reconstructive surgery?

What type/stage of breast cancer did you have?

When did you complete your treatment?

Any other family members who had cancer?

**SECTION C: INTRODUCTION TO BIOSENSORS:**

Now I’d like to talk to you about the development of biosensors.

Have you heard the term biosensor before? (if yes, where and when?)

INFORMATION B:

Biosensors are tiny devices that can be inserted into a person’s cancer tumour. They can monitor the cancer’s biology and take real-time measurements (e.g. level of oxygenation, temperature, pH level). Oxygenation is the process by which concentrations of oxygen increase within a tissue. This information can then be used to individualise radiotherapy treatment.

Biosensors that can be used routinely do not yet exist. **We are not asking you to test one**. They are currently being developed by cancer specialists and engineers in Edinburgh. One or more would be inserted by a cancer specialist into the cancer. It is hoped that biological information (e.g. levels of oxygenation in the cancer) captured by the biosensors will be able to tell clinicians (i) which regions of a cancer need extra doses of radiation to eradicate cancer cells (ii) when may be the best time to treat the tumour. The main aim would be to maximise the effectiveness of radiotherapy against cancer cells. It might also identify patients unlikely to benefit from radiotherapy.

EXAMPLE: A biosensor that can monitor blood sugar levels in people with diabetes.

Is that clear to you – or do you want me to go over that again? Tell me, what immediately springs to your mind when thinking about this technology then?

Show physical models here (two smaller ones only). These are some examples to give you an idea of what the biosensor may look like.

As I explained before, the biosensor is still being developed and a process of miniaturisation will take place. The target is to make it the size of a grain of rice (show example of grains of rice).

Do you have any thoughts about the biosensor itself – about the way it looks for example? Do you think the size is acceptable?


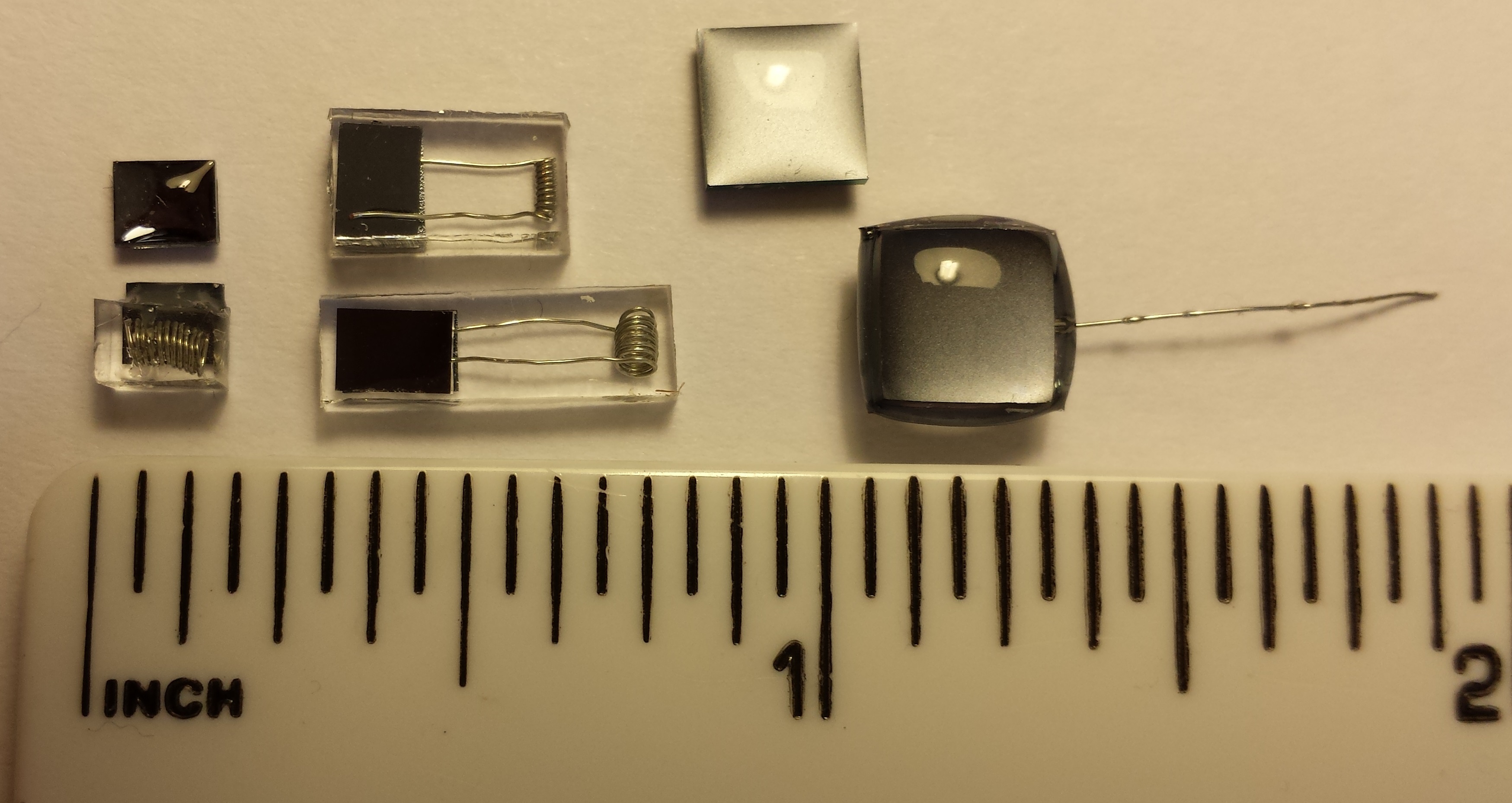


**PICTURE 1**: Different shapes and sizes

What about shape of the biosensor, do you have any preferences?

Q. In future, there may be **more than one biosensor** (perhaps 6-8) implanted in order to gain more representative measurements in different regions of a cancer. Can you say what you might have a preference for (one or more sensors)? Is there any particular reason for this?

Q. Let’s talk a bit about **method of insertion**. The biosensor(s) will be inserted by a needle. How would you feel about this?

**Tolerances of Control**

What would be the most acceptable and least acceptable from the following situations (MAKE SURE TO READ THESE OUT FOR TRANSCRIBER!):

INFORMATION C:

- The biosensor to be powered on by medical professionals and who can also switch it off.
- The biosensor to control itself and to be able to switch itself on and off in response to changes in the body.

At present our research team think the biosensor would be stationary embedded within the cancer tumour. It could be inserted before surgery (if you require surgery) and removed when surgery is carried out. How would you feel about having a biosensor implanted in such a way?

If you don’t require surgery, it might remain in the body permanently. How would you feel about this?

Use medical illustrations to show different power options here

In relation to **powering the biosensor**, there are two options: it could either be powered remotely via wireless power transfer or using wires. If the biosensor had to have wires coming out of it in order to supply power, would that change how you felt about the sensor? Do you have any preference in terms of how the device is powered (select from medical illustrations- power options)? Why? Would you prefer the device to have wires/no wires coming out of it? Would it make a difference if the wires were hidden under the surface of the skin or were visible on the surface of the skin?

Q. **Duration** of biosensor in the body. How long would be an acceptable time to have the biosensor inside your body? (Prompt 24-48 hours, 1 week, 1 month, 1 year or indefinitely)

Use medical illustrations to show data transmission options here

**Data transmission**

There are a couple of options how data could be transmitted from the sensor to the data transceiver. Data transmission could either be through a wire or wirelessly (similar to power options). Which option do you prefer (select from medical illustrations- data transmission options), and why?

Q. Thinking about the transmission of information - What would be preferable – for the sensor to be able to transmit data:

1. for a short space of time and over a short distance only (in the clinic?) to a receiver that captures the data
2. To be able to transmit to the cancer centre from home (one off)?
3. To be able to transmit data to the cancer centre from home on a regular basis?

Probe: What are your views on **data security**? Any concerns? (during transmission and how it is stored)

If participant chooses different options in power and data transmission, check why (show them different cards with medical illustrations).

Q. There is a slim chance that the sensor could **do damage to the body**. It is an extra invasive procedure and there is always the risk of infection or an allergic reaction. Is that a risk you would take?

Q. **Thinking about the risks** again – there is an even smaller chance that the sensor could cause an ‘embolism’ that is, it could block an artery or travel to the lung causing a blockage, potentially leading to a person’s death. While the risks of a serious complication may be very small, is that a risk you would be willing to take?

**Future Uses (Drug Delivery)**

Q. It is possible that in the future, that the same biosensors could also be used to deliver drugs. Tiny wells in the device could hold small quantities of anti cancer drugs. These could then be released directly into the tumour. Such smart devices might reduce he side effects of anti-cancer drugs (chemotherapy). This extends the medical value of biosensors. How do you feel about this? What are your views on this?

**GOVERNANCE**

If it was up to you, who do they think should develop and control the biosensors? (i.e. University researchers, private companies, NHS)

- Who do you think should ‘own’ the biosensor? Does it ‘belong’ to you? Is it part of you? (PROBE; before implantation, when it’s working inside the body? When it is no longer working/following completion of treatment? PROBE reasons for answer).

Development of the biosensor will be in compliance with regulatory guidelines: The biosensor is going to be regulated to make sure it is safe, it will be thoroughly tested and undergoing clinical trials.

- How important do you think it is for this technology to be developed? Should we be trying to speed up the process (as long as it is safe)?

**To finish: BENEFITS and RISKS:**

- Overall, how likely are you to allow a biosensor such as this to be implanted?
- Is there anything that would put you off (try and get them to rank their concerns if they have any ….Patients are likely to be extremely supportive of medical progress).

Anything that we have missed?

Is there anything else you would like to say?

Any other comments or questions?
