# Supplementary material for: Horizon scanning implanted biosensors in personalising breast cancer management: First pilot study of breast cancer patients views
Source: Health Sci Rep. 2018 Mar 15;1(4):e30. doi: 10.1002/hsr2.30 (PMC6266376; doi:10.1002/hsr2.30)
Supplement: Supplementary file 2 — Appendix S2. Supporting information item [file HSR2-1-e30-s002.doc]

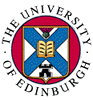

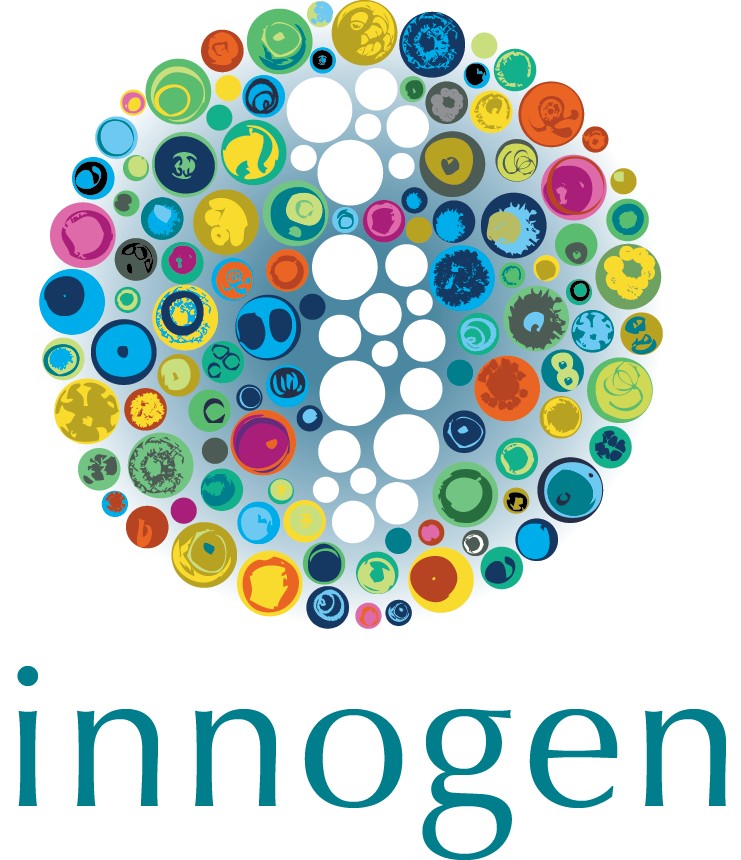


INNOGEN CENTRE

Science, Technology and Innovation Studies

The University of Edinburgh

**31^st^ March 2014** Old Surgeons Hall

High School Yards

Edinburgh EH1 1LZ

**PARTICIPANT INFORMATION SHEET (PIS) BIOS V.4** Tel. 0131 650 6389 Fax. 0131 651 4278

Theresa.ikegwuonu@ed[.ac.uk](http://www.innogen.ac.uk/)

**Seeking your views about future monitoring and personalizing of cancer treatment**

We are inviting you to take part in a research project about your views on future ways of monitoring and improving cancer treatment. These monitors (called biosensors) are tiny devices that are at a very early stage of development in research. However, if once developed, they were found to be safe and effective, one or more might be inserted into a patient’s cancer at some time in the future. They will be designed to measure the cancer’s biological activity in real time. This information has the potential to individualise radiotherapy treatment and improve cure rates. WE ARE NOT ASKING YOU TO TEST ONE OF THESE MONITORS (biosensors), and we are very grateful for your thoughts.

My name is Theresa Ikegwuonu and along with my colleague Gill Haddow, we are social science researchers working at The University of Edinburgh. We have talked about this technology with men who are in recovery from treatment of prostate cancer and we now would like to talk with 30 other individuals like yourself who have had their cancer removed and undergone radiotherapy treatment for breast cancer.

Before you decide whether or not to take part, it is important for you to understand why we are doing this research and what it will involve. Please read this leaflet carefully and, if you want, discuss it with others. This leaflet tells you the purpose of the project and what will happen if you decide to take part, and gives you more detailed information about the running of the project. Please ask us if there is anything that is not clear or if you would like more information – our details are at the top. Finally, thank you for considering our invitation.

***WHY IS THIS STUDY BEING DONE and WHY ME?***

Biosensors are tiny devices currently being developed in order to assess, in real time, the cancer environment. Such cancers can be effectively treated with radiotherapy. However, there are variations in response to treatment. It is hoped that these biosensors will be able to monitor the cancer and use this information to individualise radiotherapy treatment and to maximise the killing of cancer cells.

So this study is being done to explore the views of a variety of patients with early breast cancer who have received radiotherapy. This is why we have approached you. We are working with cancer specialists at the Western General Hospital, Edinburgh who are especially interested in what you think about ‘biosensors’. Although we are working in partnership with your consultant, we will be independent of them and they will not know whether or not you were interviewed.

***WHAT WOULD PARTICIPATION INVOLVE?***

Biosensors are currently being developed by scientists and medical professionals. WE ARE NOT ASKING YOU TO TEST ONE. What we want to do now is to interview you for approximately one hour seeking your views about this future technology. We will arrange this interview at a time and at a place that suits you best. This might be in the comfort of your own home or if more convenient, we can arrange to meet at the hospital. We know that you will not have thought about biosensors before and may not have even heard the word ‘biosensor’, but we will be able to explain what they are, what they might do and show you pictures of what they might look like and what size they might be.

***DO YOU HAVE TO TAKE PART?***

No - it is entirely voluntary. It is up to you to decide whether or not to take part. If you decide not to take part, you do not have to tell us why. You can make this decision at any point. If you choose not to participate, your medical care will not be affected in any way.

***WHAT ABOUT TRAVEL EXPENSES?***

It should not cost you anything to take part in this research, but if it does we will reimburse for reasonable local travel expenses by taxi or public transport.

***CAN YOU WITHDRAW FROM THE STUDY?***

Yes. You can withdraw from the study at any time without giving a reason.

***WHAT ABOUT CONFIDENTIALITY?***

The researchers do not have access to any information about you other than that you have provided to us. You will be asked for your written permission to pass your contact details on to us. This does not mean that you have to take part. One of the researchers will contact you to explain a bit more about the study and the interview. If you are still interested we will set up a time, date and place where we can conduct the interview. With your permission, a recording of the interview will be taken because we need an accurate record of what we discuss. Once we have analysed the recording we will destroy it. We will make sure that you will not be identified. Anything you tell us will be treated confidentially. We can assure you that any reports or publications will not contain information from which you can be identified. We will be delighted to send you a short summary of the results from the study if you so wished.

**WHAT HAPPENS NEXT?**

A member of your oncology medical staff should have already approached you to make sure that you are willing for me to contact you about this study. If after reading this information sheet you would like to take part, we would like you to contact us. Please contact the researcher whose details are listed below and we will arrange a time and place that suits you best. Let us know if you require further information. Please read the ‘Consent Form’ carefully and you can bring it with you to the interview.

Dr Theresa Ikegwuonu

INNOGEN CENTRE

Science, Technology and Innovation Studies

The University of Edinburgh

Old Surgeon’s Hall, High School Yards

Edinburgh, EH 1 1LZ

Telephone (0131) 650 6389 or e-mail: [theresa.ikegwuonu@ed.ac.uk](mailto:theresa.ikegwuonu@ed.ac.uk)

or by post at above address.

If you would like to speak to someone who is independent of the study, please contact

Dr Steve Sturdy by phone (0131) 651 4741 or e-mail: [s.sturdy@ed.ac.uk](mailto:s.sturdy@ed.ac.uk)

If you would like to make a complaint about this study, please contact NHS Lothian:

NHS Lothian Complaints Team

2nd Floor, Waverley Gate

2-4 Waterloo Place

Edinburgh, EH1 3EG

Tel: 0131 465 5708.

***Finally, thank you for your interest and for taking the time to consider this invitation.***
